# Associations of air pollution with acute coronary syndromes based on A/B/AB versus O blood types: case-crossover study

**DOI:** 10.1038/s41598-024-65506-2

**Published:** 2024-06-25

**Authors:** Tomasz Bochenek, Adam Pytlewski, Daniel Bride, Bartosz Gruchlik, Michał Lelek, Małgorzata Teodorska, Michał Nowok, Krystian Wita, Katarzyna Mizia-Stec, Benjamin D. Horne

**Affiliations:** 1 First Department of Cardiology, Medical University of Silesia, Ul. Ziołowa 47, 40-635 Katowice, Poland; 2 European Reference Network for Rare, Low Prevalence, Or Complex Diseases of the Heart (ERN GUARD Heart), Amsterdam, The Netherlands; 3 Bielanski Hospital, Warsaw, Poland; 4 Intermountain Medical Center Heart Institute, Salt Lake City, UT USA; 5 Military Institute of Medicine-National Research Institute, Warszawa, Poland; 6 Public Hospital, Number 4, Gliwice, Poland; 7 Cardiovascular Institute, Stanford University School of Medicine, Stanford, CA USA

**Keywords:** Cardiology, Diseases

## Abstract

Short-term exposure to air pollutants may contribute to an increased risk of acute coronary syndrome (ACS). This study assessed the role of short-term exposure to fine particulate matter (PM_2.5_) as well as fine and coarse PM (PM_10_) air pollution in ACS events and the effect of blood groups on this phenomenon. A retrospectively collected database of 9026 patients was evaluated. The study design was a case-crossover using a conditional logistic regression model. The main analysis focused on PM_2.5_ levels with a 1 day lag until the ACS event, using threshold-modelled predictor for all patients. Secondary analyses utilized separate threshold-modelled predictors for 2–7-days moving averages and for patients from specific ABO blood groups. Additional analysis was performed with the non-threshold models and for PM_10_ levels. Short-term exposure to increased PM_2.5_ and PM_10_ levels at a 1-day lag was associated with elevated risks of ACS (PM_2.5_: OR = 1.012 per + 10 µg/m^3^, 95% CI 1.003, 1.021; PM_10_: OR = 1.014 per + 10 µg/m^3^, CI 1.002, 1.025) for all patients. Analysis showed that exposure to PM_2.5_ was associated with increased risk of ACS at a 1-day lag for the A, B or AB group (OR = 1.012 per + 10 µg/m^3^, CI 1.001, 1.024), but not O group (OR = 1.011 per + 10 µg/m^3^, CI 0.994, 1.029). Additional analysis showed positive associations between exposure to PM_10_ and risk of ACS, with 7-days moving average models stratified by blood group revealing that exposures to PM_2.5_ and PM_10_ were associated with elevated risk of ACS for patients with group O. Short-term exposures to PM_2.5_ and PM_10_ were associated with elevated risk of ACS. Short-term exposure to PM_2.5_ was positively associated with the risk of ACS for patients with A, B, or AB blood groups for a 1-day lag, while risk in O group was delayed to 7 days.

## Introduction

### Study purpose

Recent studies suggest that chronic exposure to air pollution caused by particulate matter (PM) causes, among other things, systemic oxidative stress, the development of atherosclerosis, inflammation and increased risk of coronary heart disease and death. Even short-term exposure may contribute to the development of complications of atherosclerosis and contribute to an increased risk of heart attack. These findings are inconclusive and require further investigation. This study assessed the role of short-term air pollution exposure in acute coronary heart disease events and the effect of blood groups on this phenomenon.

### Background

Exposure to increased concentrations of PM has been identified as a hazard factor of cardiovascular disease and mortality^1–5^. Long-term repeated exposure to PM has been associated with ischemic heart disease. Empirical models for the association of mortality with PM are consistent with the hypothesis that exposure to PM contributes to oxidative stress and inflammation, the development of atherosclerosis, and the risk of coronary heart disease and death^4^. Long-term exposure to PM is associated with subclinical atherosclerosis, which contributes to the progression of atherosclerotic plaques and increased susceptibility to plaque rupture. PM also intensified vasculitis^6^. Short-term exposure to PM contributes to acute ischemic heart disease. Even short-term exposure to PM can contribute to the development of inflammation and the progression of atherosclerosis, which may cause a myocardial infarction (MI), which has already been confirmed in some studies of the general population^7–12^. In addition, it is also associated with the risk of ischemic stroke^13^.

MI is characterized by a depression of the ST segment on the ECG, increased plasma viscosity, elevated levels of inflammatory markers in the blood, and changes in the autonomic function of the heart^14^. Blood group may have an influence on the development of MI. As studies show, short-term exposure to elevated PM levels is strongly associated with the risk of MI in carriers of the A allele corresponding to blood groups A, B and AB, and with a lower association in the GG genotype corresponding to blood group O^15–17^. Short-term exposure to PM may be associated with vascular changes. It may cause arterial vasoconstriction, which results in impaired vascular reactivity and endothelial function. Patients with diabetes and high blood pressure may be most at risk of this^18^. Patients after cardiac rehabilitation and with lung diseases may also react similarly^19^.

This study assessed the role of short-term exposure to PM and the patient’s blood group on the risk of an acute ischemic heart disease event. This study used a large, continuous, and unique registry of well-characterized patients who underwent coronary angiography. The increase in PM concentration resulted from Katowice’s location in a basin with a densely populated topography and a large number of factories and mines in the city. Greater complications of PM dust exist in winter compared to other seasons, with the most substantial contributions from hard coal used to heat residential buildings and, regionally, from the operation of factories and mines in the city.

The purpose of this study is to investigate the potential role of short-term exposure to PM elevations and the influence of blood type in triggering an episode of acute ischemic heart disease in patients undergoing coronary angiography.

## Methods

### Study group

In this study, a retrospectively collected database of 9225 patients has been gathered. The bioethics Committee of Medical University of Silesia adopted a position that due to the retrospective nature of the research, subject approval is not necessary, thus a waiver of informed consent was given (number of the decision: KNW/0022/KB/70/18). All methods were performed in accordance with the relevant guidelines and regulations and in keeping with the Declaration of Helsinki. Participants were recruited from among the patients from the Department of Cardiology, Medical University of Silesia, Katowice, Poland, who were seen for urgent or emergent symptoms of acute coronary syndrome (ACS).

Among the ACS events, 5282 (57%) of the patients were diagnosed with unstable angina (UA), 2608 (28%) with non-ST-elevation MI (NSTEMI), 1310 (14%) with ST-elevated MI (STEMI) and 25 patients did not receive a clear diagnosis. Among all of the patients (n = 9225), 123 had missing information about blood group (n = 9102) and those patients were excluded from study analyses. Air pollution data was also missing for dates when 76 patients had their ACS event, thus the final number of patients included in case-crossover analyses was 9026. The clinical characteristics of patients were collected between December, 2012 and December, 2017 (Table 1).

**Table 1:** Characteristics of the participants.

Feature	All patients n = 9225	Blood type A n = 3774	Blood type B n = 1714	Blood type AB n = 781	Blood type O n = 2833
$\text{Age}$(years)	67.8 ± 11.5	67.8 ± 10.3	68.3 ± 10.1	67.8 ± 11.5	68.0 ± 10.2
$\text{Sex}$(% of females)	36	35	36	36	33
$\text{Obesity}$(%)	29	29	29	29	29
$\text{Hypertension}$(%)	86	86	88	86	87
$\text{Hyperlipidemia}$(%)	62	63	62	62	64
$\text{History of heart failure}$(%)	6	8	8	6	7
$\text{History of renal failure}$(%)	10	9	8	10	9
$\text{Family history of early CHD}$(%)	21	20	19	21	19
$\text{Currently smoking}$(%)	19	17	17	19	16
$\text{Smoking in the past}$(%)	46	50	48	46	51
$\text{IFG}$(%)	1	1	1	1	2
$\text{IGT}$(%)	2	2	2	2	2
$\text{Diabetes type I}$(%)	1	1	1	1	1
$\text{Diabetes type II}$(%)	33	29	32	33	31
$\text{UA}$(%)	57	58	57	57	58
$\text{NSTEMI}$(%)	29	28	28	29	29
$\text{STEMI}$(%)	14	15	15	14	13

Data are presented as mean ± SD, number of patients or percentages.

SD, standard deviation; CHD, coronary heart disease; IFG, impaired fasting glycemia; IGT, impaired glucose tolerance; UA, unstable angina; NSTEMI, non-ST-elevation myocardial infarction; STEMI, ST-elevated myocardial infarction.

### Study area and pollution data

Katowice is the biggest city in Silesia, with over 300,000 people, according to the data from 2014, provided by Polish Central Statistical Office. The city is situated on the Silesian Upland with a large number of factories and mines in the city. This translates to poor air quality, especially in winter, with short-term high concentrations of PM_2.5_ and PM_10_ occurring periodically especially during high pressure weather patterns. The climate is moderately warm and average humidity is around 72%, with the highest humidity in January (88%) and the lowest in August (66%).

**Figure 1:**
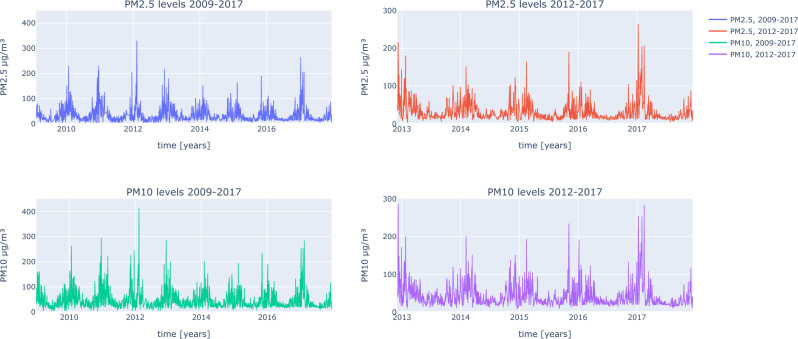
PM_2.5_ and PM_10_ levels from the whole available period (3287 days) and the period when patients characteristics were collected (2192 days), number of patients: 9225.

Air pollution data was obtained from the Chief Inspectorate of Environmental Protection (GIOS, Poland), using a central Air Quality Measurement Station, located on Kossutha Street, Katowice, Poland, with PM_2.5_ and PM_10_ available for the full study period with few daily exceptions, having been measured starting in January 1, 2009, and collected through December 31, 2017. This was the only air quality measurement stations which was used in the study. It was the closest station to the First Department of Cardiology at the Silesian Hospital in Katowice—a hospital from which participants of the study were recruited. Straight line distance between those two objects is 6.4 km. The station is located near several mines, including: the Wujek mine (2.6 km), the Muricki-Staszic mine (6.8 km) and Mysłowice-Wesoła mine (12.5 km) PM_2.5_ and PM_10_ levels were measured hourly, 24 h a day. The data that GIOS provided were daily means of those values and those were used in the study (Fig. 1). From PM_2.5_ and PM_10_ data, 11% and 3% of the values were missing, respectively. From the period where patient characteristics were collected, 8% and 3% of the PM_2.5_ and PM_10_ data was missing, respectively. Days with missing values were not included in the analysis.

Weather data was obtained from the Institute of Meteorology and Water Management (IMGW, Poland), using a weather station located on Pyrzowice Airport, Katowice, Poland. Weather data (mean daily temperature, humidity, wind speed, and nebulosity) were only available for the years 2012–2015, but were used in subset analyses of the reduced sample size to evaluate confounding of the primary results. A change in the regression beta-coefficients for PM_2.5_ and PM_10_ variables of > 10% when the four weather variables were added to the model was considered to indicate confounding. Weather data analysis has been included in supplementary materials.

### Statistical analysis

The case-crossover study design is an observational study where each subject serves as their own control. This analytic method utilizes the date of an event and compares the ecological exposure to air pollution levels on that day, days leading up to it, or a moving average of multiple days prior to the event to levels of the exposure in the same time frame relative to control days on which the event did not happen. Control days are chosen to be in the same month and on the same day of the week as the event, but in the other weeks of that month when the event did not happen. This design allows for the control of all patient-specific factors as well as the seasonally-variant factors related to weather and weekly-variant factors connected to human activities and cultural norms such as weekday versus weekend work and recreation. Case-crossover studies are widely used in situations where a short-term, potential risk factor could trigger an acute reaction or disease^20^.

The statistical modelling approach used for such studies typically is conditional logistic regression. This model performs comparisons between the same subject based on air pollution levels, but allows for adjustment for daily-variant factors such as temperature, humidity, and barometric pressure.

We performed a case-crossover study, using conditional logistic regression models, analysing the influence of PM_2.5_ and PM_10_ on the risk of the ACS event. The chosen referent periods in the study were on the same day of the week in the same month of the year. The main analysis focused on PM_2.5_ levels with a 1-day lag until the ACS event, using a threshold-modelled variable for all patients. Secondary analyses utilized separate threshold-modelled variables for patients from specific ABO blood groups in which people with A, B, or AB blood type were analyzed separately from those with O blood type. Additional analysis was performed with non-threshold models for PM_10_ levels and with calculated averages of PM_2.5_ and PM_10_ values (2, 3, and 7 days moving averages (MA)). Thresholds for part of the models were set to 35 µg/m^3^ and 150 µg/m^3^, for PM_2.5_ and PM_10_, respectively. Values below the thresholds were set to 0, and values above the thresholds were shifted down by 35 and 150, respectively. The thresholds choice was based on US Environmental Protection Agency (EPA) thresholds for PM_2.5_ and PM_10_. Additional analysis of models using WHO thresholds was included in supplementary materials. Odds ratios (OR) with 95% confidence intervals (95% CI) and *p* values were calculated for each model. Adjustment for temperature, humidity, and barometric pressure was not possible due to a lack of data on these parameters. Statistical significance level was set to 0.050 (*p* <  = 0.050). Calculations were performed using R software.

### Ethical approval and consent to participate

Not applicable. Bioethics Committee of Medical University of Silesia adopted a position that due to the retrospective nature of the research paper approval of the Committee is not necessary, number of the decision: KNW/0022/KB/70/18. All methods were performed in accordance with the relevant guidelines and regulations including the Declaration of Helsinki.

## Results

Baseline characteristics of the study population (n = 9225) are shown in Table 1 .

**Figure 2:**
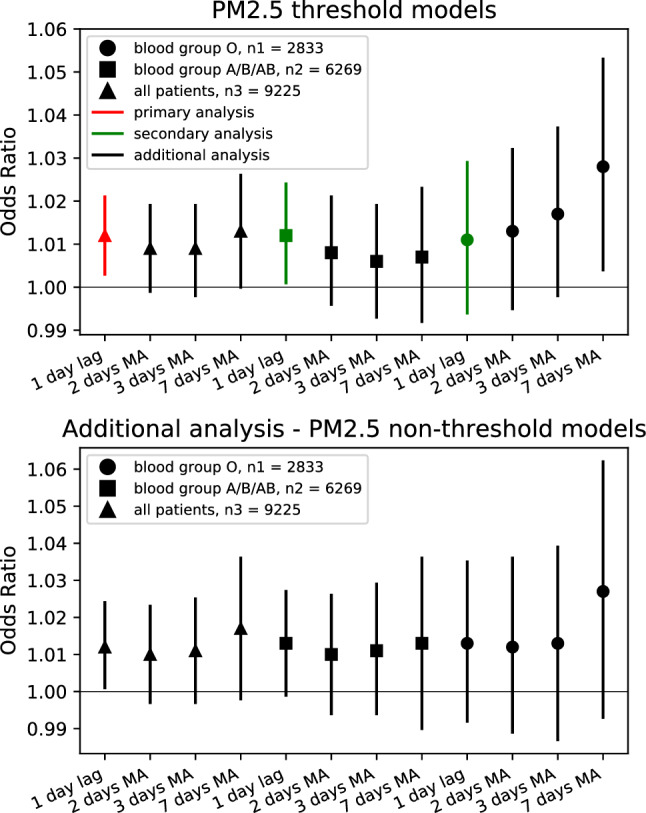
Forest plots of the influence of PM_2.5_ concentration on ACS events. Odds ratios with 95%CIs for ACS events per 10 µg/m^3^ increase in PM_2.5_. Threshold was set to 35 µg/m^3^ for threshold-based models. CI, confidence interval; MA, moving average; n1, number of patients with blood group O (2833); n2, number of patients with blood group A, B and AB (6269); n3, total number of patients (9225).

Statistically significant models analyzing the association of PM_2.5_ with ACS outcomes were: the primary threshold-based model for all of the patients had OR = 1.012 per + 10 μg/m^3^, (95% CI 1.003–1.021; *p* = 0.013) for PM_2.5_ levels above 35 μg/m^3^ at a lag of 1 day (Fig. 2 ). When the threshold was ignored, a non-threshold model for all patients also remained significant (Table 2). Threshold-based models were also significant for all of the patients for 7-days MA of PM_2.5_, while 2-days MA and 3-days MA results were weak non-significant trends (Table 2). In non-threshold models, all of the 2-days MA, 3-days MA, and 7-days MA results also had non-significant results (Table 2).

**Table 2:** Results of conditional logistic regression for PM_2.5_.

Blood type	1-day lag		2-days MA		3-days MA		7-days MA	
	OR (95% CI)	*p* value	OR (95% CI)	*p* value	OR (95% CI)	*p* value	OR (95% CI)	*p* value
*Threshold-modelled (> 35 µg/m*^*3*^*)*								
All	1.012 (1.003–1.021)	0.013	1.009 (0.999–1.019)	0.08	1.009 (0.998–1.019)	0.12	1.013 (1.000–1.026)	0.047
A/B/AB	1.012 (1.001–1.024)	0.032	1.008 (0.996–1.021)	0.20	1.006 (0.993–1.019)	0.38	1.007 (0.992–1.023)	0.35
O	1.011 (0.994–1.029)	0.20	1.013 (0.995–1.032)	0.16	1.017 (0.998–1.037)	0.08	1.028 (1.004–1.053)	0.021
*Non-threshold*								
All	1.012 (1.001–1.024)	0.038	1.010 (0.997–1.023)	0.15	1.011 (0.997–1.025)	0.13	1.017 (0.998–1.036)	0.09
A/B/AB	1.013 (0.999–1.027)	0.08	1.010 (0.994–1.026)	0.23	1.011 (0.994–1.029)	0.21	1.013 (0.990–1.036)	0.29
O	1.013 (0.992–1.035)	0.22	1.012 (0.989–1.036)	0.32	1.013 (0.987–1.039)	0.33	1.027 (0.993–1.062)	0.12

Data presented in brackets are 95% CI.

Values have been scaled so that each term’s OR and 95% CI are representative of an increase of + 10 µg/m^3^.

For threshold-modelled predictors all PM_2.5_ values ≤ 35 µg/m^3^, were set to 0.

MA, moving average; OR, odds ratio.

**Figure 3:**
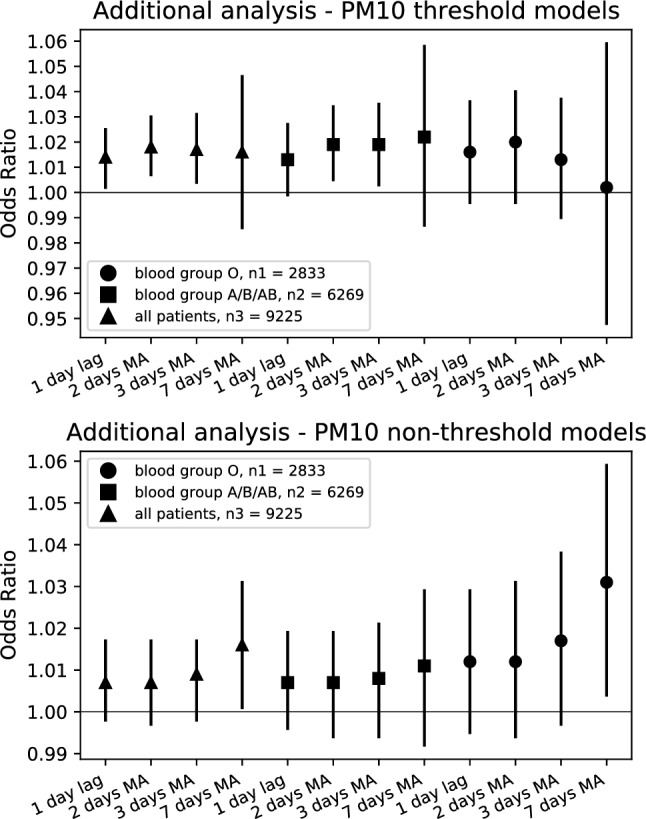
Forest plots of the influence of PM_10_ concentration on ACS events. Odds ratios with 95%CIs for ACS events per 10 µg/m^3^ increase in PM_10_. Threshold was set to 150 µg/m^3^ for threshold-based models. CI, confidence interval; MA, moving average; n1, number of patients with blood group O (2833); n2, number of patients with blood group A, B and AB (6269); n3, total number of patients (9225).

Statistically significant models analyzing PM_10_ on patients health (Fig. 3) included: the threshold-based modelling for all patients using 1 day lag (OR = 1.014 per + 10 μg/m^3^, 95% CI 1.002, 1.025; *p* = 0.017), and the 2-days MA and 3-days MA, but not the 7-days MA (Table 3). Interestingly, the results for PM_10_ were not significant in non-threshold models, except for the 7-days MA (Table 3).

**Table 3:** Results of conditional logistic regression for PM_10_.

Blood type	1-day lag		2-days MA		3-days MA		7-days MA	
	OR (95% CI)	*p* value	OR (95% CI)	*p* value	OR (95% CI)	*p* value	OR (95% CI)	*p* value
*Threshold-modelled (> 150 µg/m*^*3*^*)*								
All	1.014 (1.002–1.025)	0.017	1.018 (1.007–1.030)	0.002	1.017 (1.004–1.031)	0.009	1.016 (0.986–1.046)	0.294
A/B/AB	1.013 (0.999–1.027)	0.062	1.019 (1.005–1.034)	0.007	1.019 (1.003–1.035)	0.017	1.022 (0.987–1.058)	0.222
O	1.016 (0.996–1.036)	0.118	1.020 (0.996–1.040)	0.054	1.013 (0.990–1.037)	0.262	1.002 (0.948–1.059)	0.952
*Non-threshold*								
All	1.007 (0.998–1.017)	0.123	1.007 (0.997–1.017)	0.172	1.009 (0.998–1.017)	0.099	1.016 (1.001–1.031)	0.036
A/B/AB	1.007 (0.996–1.019)	0.200	1.007 (0.994–1.019)	0.299	1.008 (0.994–1.021)	0.259	1.011 (0.992–1.029)	0.259
O	1.012 (0.995–1.029)	0.162	1.012 (0.994–1.031)	0.197	1.017 (0.997–1.038)	0.099	1.031 (1.004–1.059)	0.024

Data presented in brackets are 95% CI.

Values have been scaled so that each term’s OR and 95% CI are representative of an increase of + 10 µg/m^3^.

For threshold-modelled predictors all PM_10_ values ≤ 150 µg/m^3^, were set to 0.

MA, moving average; OR, odds ratio.

Of further interesting, when evaluating the associations of PM_2.5_ and PM_10_ on ACS in strata defined by ABO blood type, those patients with A, B, or AB blood types mirrored the 1-day lag results for the threshold model, with a significant association of PM_2.5_ with ACS (*p* = 0.032, see Table 2). Patients with O blood type, though, did not have the immediate risk at the 1-day lag or for 2-days MA or 3-days MA, although after a week of elevated PM_2.5_ the 7-days MA was associated with ACS for O blood type (Table 2). The non-threshold results for all blood type substrata were not significant (Table 2). A similar pattern of association was seen for PM_10_ based on blood type (Table 3).

Interestingly, analysis results showed the risk from short term exposure to increased PM_2.5_ for A, B and AB blood groups was similar to a previous study in a US population, but found no significant relation between PM_2.5_ and patients with blood type O^21^. Analysis results with blood group O here suggest that cumulative short-term (7 days MA) exposure of PM_2.5_ and PM_10_ could have harmful impact on those patients rather than the immediate effect suggested for A, B and AB blood groups.

In subset analyses adjusting for weather data, reduction in regression beta-coefficients were minimal (< 10%) in almost all analyses with all four weather variables included in regression modeling, including for the primary lag 1 and for the 2-days moving average lag for PM_2.5_ and PM_10_ analyses. This was reflected in the odds ratios were minimal where their change from the base models that only entered the pollution variables to models entering all four weather variables (Supplemental Table S3). Temperature and humidity had some influence on results, especially for PM_2.5_, but these effects were reversed by adjustment for wind and nebulosity.

## Discussion

The results of this study show that short-term exposure to increased PM_2.5_ and PM_10_ levels is associated with increased risk of ACS. In the main analysis threshold-based modelling with 1 day lag PM_2.5_ concentration showed significant increase of risk for all the patients. The same association was observed in the secondary analysis for patients with A, B, or AB blood group. In additional analysis, threshold-based models for all the patients and the group with blood type O using a 7 days MA of PM_2.5_, and a non-threshold model for all the patients using 1 day lag PM_2.5_ concentration also had significant results. Significant models analyzing PM_10_ included: threshold-based models for all patients using 1 day lag, 2 days MA and 3 days MA; threshold-based models for patients with blood groups A, B or AB using 2 days MA and 3 days MA; and non-threshold models for all the patients and blood type O patients using the 7 days MA.

Similar results for ACS but without blood group analysis have been reported in systematic reviews^22–26^. de Bont et al.^22^ in an umbrella review of ambient air pollution and cardiovascular diseases from 2022 indicated that 4 out of 4 reviews^23–26^ showed dependence between short-term PM_2.5_ and PM_10_ exposure and increasing MI mortality rates, hospital admissions, and/or ischemic heart diseases. In one of those 4 reviews, Mustafić et al.^23^ focused on MI risk estimation and included 34 studies from locations on every continent except Africa, with 17 also using the case-crossover model and the rest being time-series studies. They showed in a meta-analysis that higher MI risk was associated with elevated short-term exposure to PM_2.5_, PM_10_, nitrogen dioxide, sulfur dioxide, and carbon monoxide^23^. Cai et al. included and analyzed 33 studies, where 25 of them were focused on increased hospitalization rates and 8 on increased mortality. Studies were conducted in Asia (China, Taiwan and Japan), Europe (Sweden, France, England and Wales, Italy and Spain), America (Canada, US, Brazil), Australia and Oceania (Australia and New Zealand). Most of them used the case-crossover design and reported from a meta-analysis that both PM_2.5_ and PM_10_ were associated with hospitalization for MI and with death due to MI^24^. Luo et al. reviewed the risk of MI from 31 studies of 4 geographic regions (Asia (Taiwan, Japan, China), America (US, Brazil, Canada), Europe (France, Germany, Finland, Italy, Netherlands, Spain, England and Wales, Sweden), Australia) and documented by meta-analysis the existence of a significant association of both short-term elevations in PM_2.5_ and PM_10_ with MI, with higher odds ratios for PM_2.5_, but they did not differentiate whether the events were fatal or non-fatal MIs^25^. Farhadi et al. reviewed studies analyzing PM_2.5_ and risk of hospitalization for MI, with 26 studies being included in meta-analyses, where analyses were either case-crossover or time-series study, and focusing on PM_2.5_ reported significant association of short-term elevation in air pollution with MI^26^. The lag used in studies included in the reviews varied mostly from 0 to 7 days—same range of lags have been analyzed in our study.

The number of articles analyzing short-term PM exposure and an ACS risk relation with the additional factor of ABO blood group was significantly smaller. No systematic review of the potential interaction of blood group with air pollution was found. ABO blood group is the only genetic locus that is validated as a risk factor for MI in international genetic association studies of people with coronary disease^27^. Some studies claim that other genetic loci are associated with MI, but those loci actually differentiate between presence and absence of coronary disease where MI is the most severe consequence of atherosclerosis but in almost all situations coronary disease must be present for MI to occur^27^. Horne et al. indicated that carriers of the ABO rs687289 A allele had stronger association between short-term exposure to increased PM_2.5_ and risk of ACS^21^. Huang et al. found that a particularly strong positive association exists between ambient air pollution and costs of hospitalization for MI patients with blood group B^28^. Suadicani et al. further analyzed risk of ischemic heart disease (IHD) risk among men with long-term occupational exposure to airborne pollutants. That study showed that men with blood group O had increased risk of IHD, whereas men with other blood groups didn’t have similar significant dependency^29^. Three different studies indicated three blood groups (A, B, and AB) were potential risk factors for ACS, MI, and IHD. The present study’s main analysis showed results similar to the Horne and Huang studies^21,28^. Associations were found between exposure to PM_2.5_ and PM_10_ and elevated risk of ACS for patients with group O only in 7 days MA models, but not in other models. A potential explanation could be that people with blood group O are prone to ACS after longer exposure or greater cumulative exposure to air pollution.

This study could have some potential limitations. The retrospective study design implies limitations such as risk of recall and observer bias, and involves a difficulty in establishing cause and effect as well as issues with reliability of the data. Despite a reasonable number of patients, the area of the study was not vast. The broader part of Poland should be assessed to gain more information about the influence of air pollution and blood group on ACS risk. The case-crossover study design was very helpful in reducing potential bias when acquiring information about the influence of blood group, but this design limited the study’s ability to discover other risk factors for ACS since most factors were internally matched by design. While weather data were not available for the whole period of the study, adjustment in a subset of patients from 2012 to 2015 indicated minimal to no confounding by weather factors on PM_2.5_ or PM_10_ associations with ACS outcomes. This suggests that the primary results of the study for the overall population are reliable, but caution should be taken in interpretation since weather data were not available for the whole study period. Subanalyses by blood type should be further validated in other populations with full adjustment for weather variables.

A strength of our study is that potential ABO risk groups being connected with short-term influence of PM on ACS risk is a novel topic, with only a few other reports. Another strength is that there are not many studies analyzing air quality as a risk factor for ACS that have been performed in Eastern Europe.

## Conclusions

Short-term exposure to PM_2.5_ and PM_10_ was associated with elevated risk of ACS. Short-term 1-day lag exposure to PM_2.5_ positively associated with the risk of ACS across all patients, as well as when stratification focused on the blood group of those with A, B, or AB blood type. Additional analysis showed a positive association between exposure to PM_10_ and the risk of ACS. Finally, prolonged exposure to PM_2.5_ and PM_10_ (modeled as the 7 days moving average for the independent variable) was associated with higher risk of ACS in patients with blood group O. More significant results were obtained using threshold models than no-threshold analyses. Additional research on a broader spectrum of patients should be performed to validate and expand on these findings.

## Supplementary Information

Supplementary Figure S1.

Supplementary Figure S2.

Supplementary Figure S3.

Supplementary Information 4.

Supplementary Table S1.

Supplementary Table S2.

Supplementary Table S3.

## Data Availability

The data underlying this article will be shared on reasonable request to the corresponding author.

## Abbreviations

PM_2.5_: Particulate matter with particles that are 2.5 microns or less in diameter
PM_10_: Particulate matter with particles that are 10 microns or less in diameter
ACS: Acute coronary syndrome
UA: Unstable angina
NSTEMI: Non-ST-elevation myocardial infarction
STEMI: ST-elevated myocardial infarction
OR: Odds ratio
CI: Confidence interval
MA: Moving average
MI: Myocardial infarction
