# Author Correction: Associations of air pollution with acute coronary syndromes based on A/B/AB versus O blood types: case-crossover study

**DOI:** 10.1038/s41598-024-72914-x

**Published:** 2024-09-18

**Authors:** Tomasz Bochenek, Adam Pytlewski, Daniel Bride, Bartosz Gruchlik, Michał Lelek, Małgorzata Teodorska, Michał Nowok, Krystian Wita, Katarzyna Mizia-Stec, Benjamin D. Horne

**Affiliations:** 1 First Department of Cardiology, Medical University of Silesia, Ul. Ziołowa 47, 40‑635 Katowice, Poland; 2 European Reference Network for Rare, Low Prevalence, Or Complex Diseases of the Heart (ERN GUARD Heart), Amsterdam, The Netherlands; 3 Bielanski Hospital, Warsaw, Poland; 4 Intermountain Medical Center Heart Institute, Salt Lake City, UT USA; 5 Military Institute of Medicine-National Research Institute, Warszawa, Poland; 6 Public Hospital, Number 4, Gliwice, Poland; 7 Cardiovascular Institute, Stanford University School of Medicine, Stanford, CA USA

Correction to: *Scientific Reports*10.1038/s41598-024-65506-2, published online 25 June 2024.

The original version of this Article contained an error in the spelling of the author Katarzyna Mizia-Stec, which was incorrectly given as Katrzyna Mizia Stec.

The original Article has been corrected.

