# Supplementary figures and images for: Associations of air pollution with acute coronary syndromes based on A/B/AB versus O blood types: case-crossover study
Source: Sci Rep. 2024 Jun 25;14:14580. doi: 10.1038/s41598-024-65506-2 (PMC11199661; doi:10.1038/s41598-024-65506-2)

**Figure S1.** PM2.5, PM10 and weather data from Dec 2012 to Dec 2015.

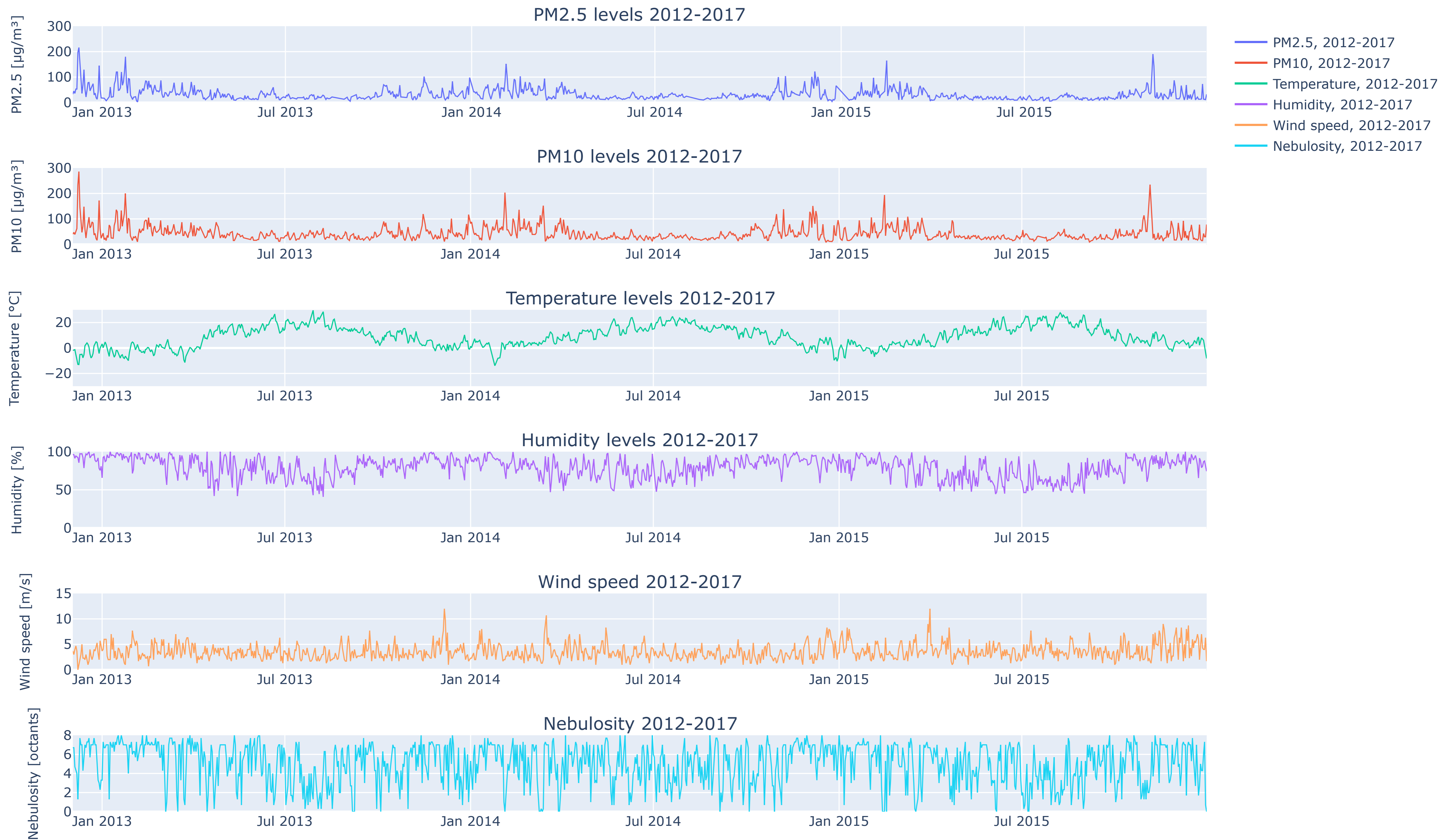

Supplement: Supplementary file 1 — Supplementary Figure S1. [file 41598_2024_65506_MOESM1_ESM.pdf]

**Figure S2.** PM2.5 and weather data, correlations

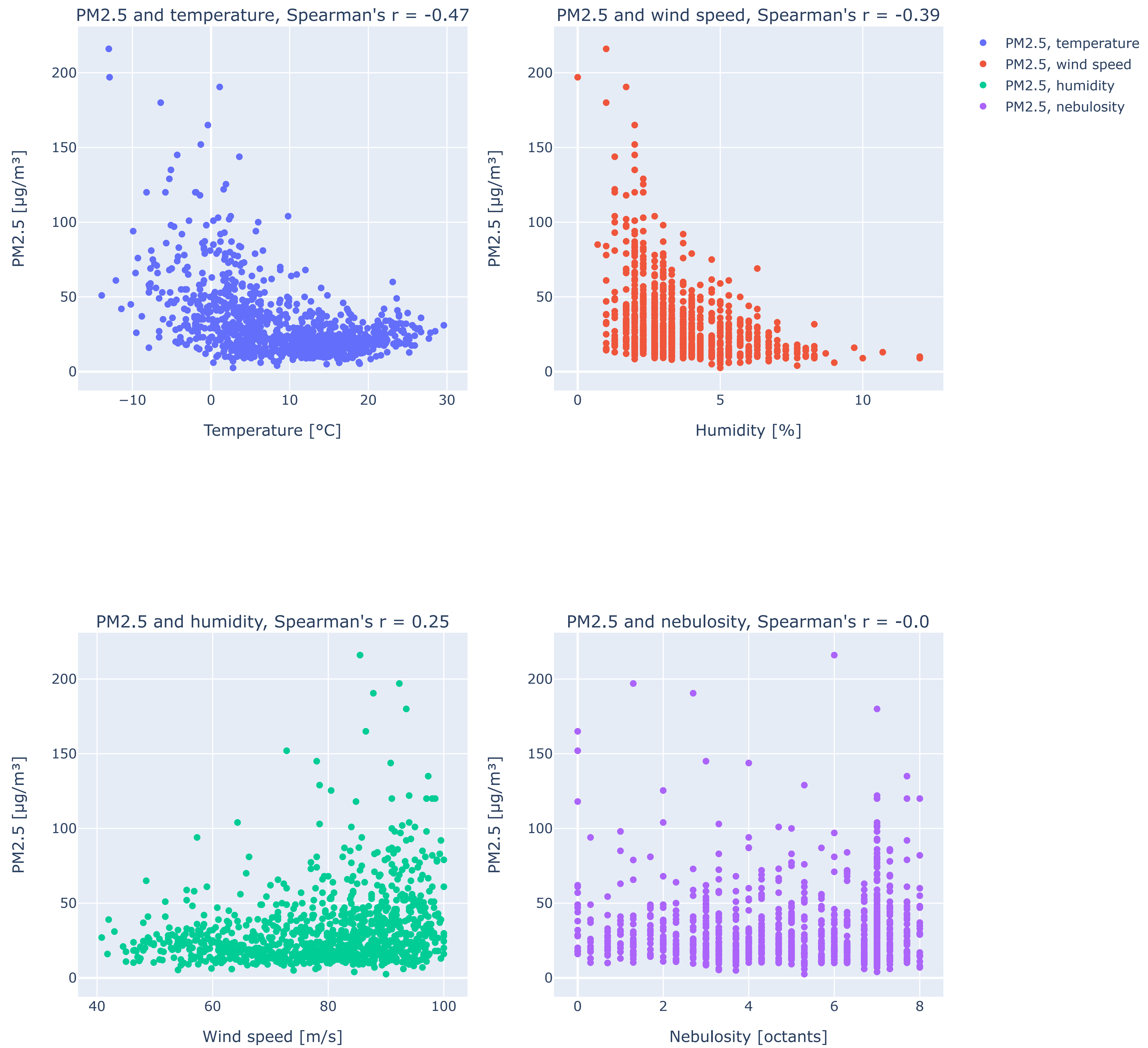

Supplement: Supplementary file 2 — Supplementary Figure S2. [file 41598_2024_65506_MOESM2_ESM.pdf]

**Figure S3.** PM10 and weather data, correlations

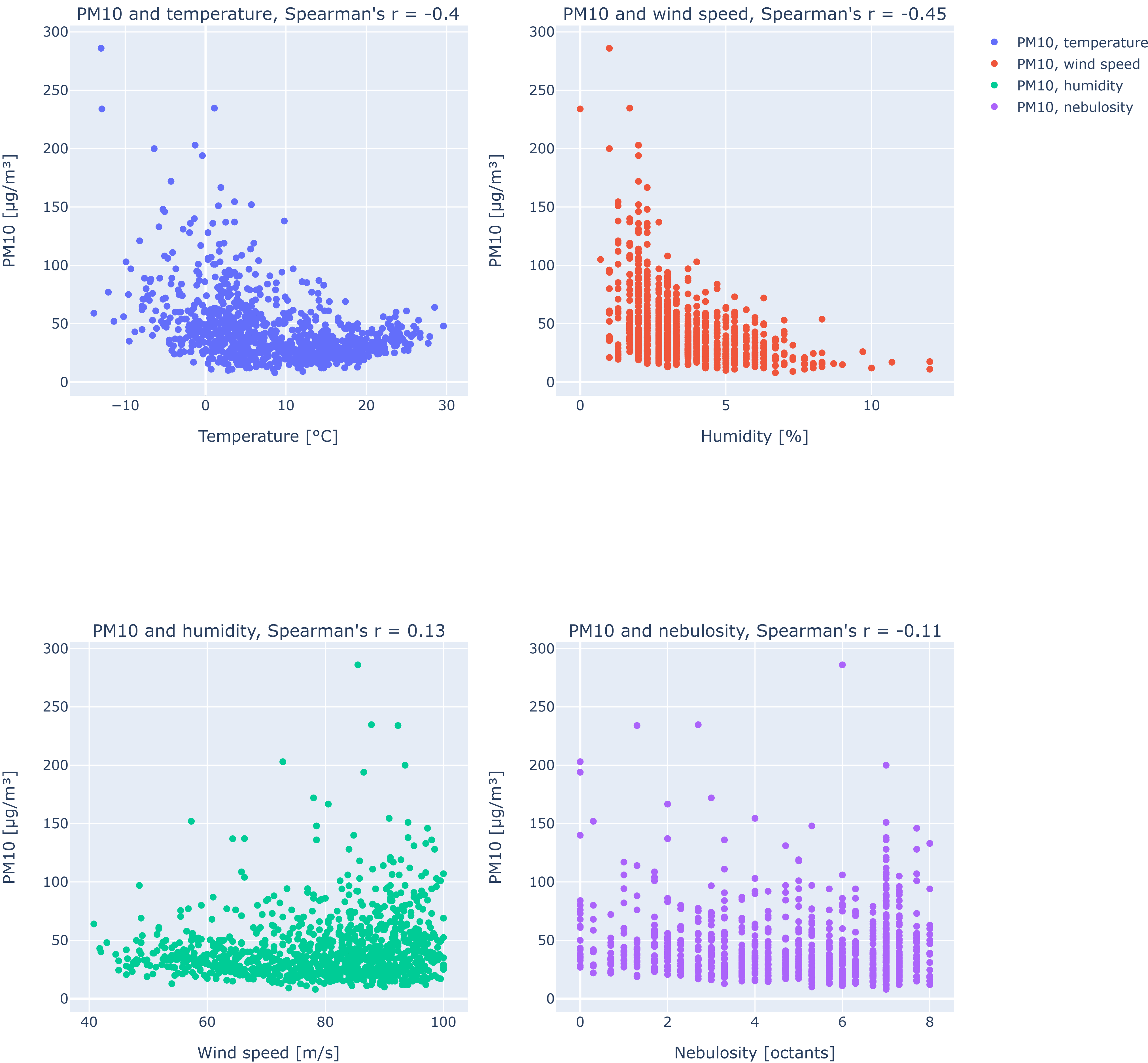

Supplement: Supplementary file 3 — Supplementary Figure S3. [file 41598_2024_65506_MOESM3_ESM.pdf]
