# Supplementary material for: Associations of air pollution with acute coronary syndromes based on A/B/AB versus O blood types: case-crossover study
Source: Sci Rep. 2024 Jun 25;14:14580. doi: 10.1038/s41598-024-65506-2 (PMC11199661; doi:10.1038/s41598-024-65506-2)
Supplement: Supplementary file 4 — Supplementary Information 4. [file 41598_2024_65506_MOESM4_ESM.docx]

**Supplementary materials**

Supplementary materials contain Table S1 which presents results of conditional logistic regression for PM2.5 on each day of the first week after air pollution elevation, Table S2 which includes results of conditional logistic regression for PM2.5 and PM10 when modeled using WHO thresholds, Figure S1 which present pollution and weather data and Figures S2, S3 which show correlations between PM2.5 and PM10 and weather data. Analyzing models with different lags (0-day up to 6-day) we did not found statistically significant models except for 1-day lag predictors described in the main article (Table S1). Models created with the US Environmental Protection Agency (EPA) thresholds for PM2.5 and PM10 proved to be more significant than models created with WHO threshold. Models analyzing PM10 with 50µg/m³ threshold showed significant correlation between prolonged exposure to PM10 and risk of ACS for all population as well as people with blood group O (Table S2).

Weather data consisted of temperature [°C], humidity [%], wind speed [m/s] and nebulosity [octants]. According to Figure S2 there was moderate negative correlation between PM2.5 levels and temperature, as well as wind speed. Weak positive correlation between PM2.5 levels and humidity was also found. Similar results were found with PM10 levels, with additional weak positive correlation between PM10 and nebulosity (Figure S3). Influence of weather conditions combined with the air pollution on ACS in Silesia’s population seems to be an interesting topic for further scientific research.
