# Supplementary material for: Associations of air pollution with acute coronary syndromes based on A/B/AB versus O blood types: case-crossover study
Source: Sci Rep. 2024 Jun 25;14:14580. doi: 10.1038/s41598-024-65506-2 (PMC11199661; doi:10.1038/s41598-024-65506-2)
Supplement: Supplementary file 5 — Supplementary Table S1. [file 41598_2024_65506_MOESM5_ESM.docx]

| **Table S1. Results of conditional logistic regression for PM_2.5_ on each day of the first week after air pollution elevation.** | | | | | | | | | | | | | | | | | | | | |
| --- | --- | --- | --- | --- | --- | --- | --- | --- | --- | --- | --- | --- | --- | --- | --- | --- | --- | --- | --- | --- |
|  |  |  |  |  | | |  | |  | |  |  | | |  | | | | | |
|  | **0-day lag** | | **1-day lag** | | | **2-day lag** | | | | **3-day lag** | | | | **4-day lag** | | | **5-day lag** | | **6-day lag** | |
| **Blood type** | **OR (95% CI)** | **p value** | **OR (95% CI)** | | **p value** | **OR (95% CI)** | | **p value** | | **OR (95% CI)** | | | **p value** | **OR (95% CI)** | | **p value** | **OR (95% CI)** | **p value** | **OR (95% CI)** | **p value** |
| **Threshold-modelled (>35 µg/m³)** | | | | | | | | | | | | | | | | | | | | |
| **All** | 1.003 (0.994 – 1.013) | 0.46 | 1.012 (1.003 – 1.021) | | 0.013 | 1.001 (0.991 – 1.011) | | 0.87 | | 1.005 (0.995 – 1.015) | | | 0.30 | 1.005 (0.995 – 1.015) | | 0.32 | 1.006 (0.996 – 1.016) | 0.25 | 0.997 (0.988 – 1.007) | 0.61 |
| **A/B/AB** | 1.003 (0.992 – 1.014) | 0.59 | 1.012 (1.001 – 1.024) | | 0.032 | 0.999 (0.988 – 1.011) | | 0.92 | | 1.003 (0.992 – 1.016) | | | 0.57 | 1.004 (0.992 – 1.016) | | 0.49 | 1.003 (0.991 – 1.015) | 0.66 | 0.993 (0.981 – 1.005) | 0.25 |
| **O** | 1.004 (0.988 – 1.021) | 0.61 | 1.011 (0.994 – 1.029) | | 0.20 | 1.008 (0.990 – 1.026) | | 0.39 | | 1.009 (0.991 – 1.027) | | | 0.32 | 1.008 (0.991 – 1.026) | | 0.36 | 1.012 (0.995 – 1.030) | 0.18 | 1.005 (0.988 – 1.022) | 0.57 |
| **Non-threshold** | | | | | | | | | | | | | | | | | | | | |
| **All** | 1.007 (0.996 – 1.018) | 0.24 | 1.012 (1.001 – 1.024) | | 0.038 | 1.002 (0.990 – 1.015) | | 0.70 | | 1.009 (0.997 – 1.022) | | | 0.15 | 1.011 (0.999 – 1.024) | | 0.07 | 1.007 (0.995 – 1.020) | 0.23 | 0.997 (0.985 – 1.009) | 0.62 |
| **A/B/AB** | 1.006 (0.992 – 1.020) | 0.39 | 1.013 (0.999 – 1.027) | | 0.08 | 1.001 (0.987 – 1.016) | | 0.87 | | 1.008 (0.993 – 1.023) | | | 0.32 | 1.009 (0.994 – 1.025) | | 0.23 | 1.004 (0.989 – 1.019) | 0.58 | 0.995 (0.979 – 1.010) | 0.48 |
| **O** | 1.008 (0.988 – 1.029) | 0.42 | 1.013 (0.992 – 1.035) | | 0.22 | 1.007 (0.986 – 1.030) | | 0.50 | | 1.012 (0.990 –1.034) | | | 0.29 | 1.019 (0.996 – 1.041) | | 0.10 | 1.014 (0.993 – 1.036) | 0.19 | 1.000 (0.979 – 1.021) | 0.99 |
| • Data presented in parentheses are 95% CI.  • Abbreviations: OR – odds ratio, CI – confidence interval  • Values have been scaled so that each term's OR and 95% CI are representative of an increase of +10 µg/m³.  • For threshold-modelled predictors all PM_2.5_ values ≤ 35µg/m³, were set to 0. | | | | | | | | | | | | | | | | | | | | |
