# Supplementary material for: Associations of air pollution with acute coronary syndromes based on A/B/AB versus O blood types: case-crossover study
Source: Sci Rep. 2024 Jun 25;14:14580. doi: 10.1038/s41598-024-65506-2 (PMC11199661; doi:10.1038/s41598-024-65506-2)
Supplement: Supplementary file 6 — Supplementary Table S2. [file 41598_2024_65506_MOESM6_ESM.docx]

| **Table S2. Results of conditional logistic regression for PM_2.5_ and PM_10_ when modeled using WHO thresholds.** | | | | | | | | |
| --- | --- | --- | --- | --- | --- | --- | --- | --- |
|  |  | | | | | | | |
|  | **1-day lag** | | **2-day MA** | | **3-day MA** | | **7-day MA** | |
| **Blood type** | **OR (95% CI)** | **p value** | **OR (95% CI)** | **p value** | **OR (95% CI)** | **p value** | **OR (95% CI)** | **p value** |
| **PM_2.5_ Threshold-modelled, >25 µg/m³** | | | | | | | | |
| **All** | 1.009 (0.9996 – 1.019) | 0.059 | 1.008 (0.998 – 1.018) | 0.13 | 1.007 (0.996 – 1.018) | 0.22 | 1.008 (0.995 – 1.022) | 0.23 |
| **A/B/AB** | 1.009 (0.998 – 1.021) | 0.12 | 1.009 (0.997 – 1.021) | 0.15 | 1.009 (0.995 – 1.022) | 0.20 | 1.007 (0.991 – 1.024) | 0.38 |
| **O** | 1.012 (0.994 – 1.029) | 0.20 | 1.006 (0.988 – 1.024) | 0.52 | 1.005 (0.986 – 1.025) | 0.59 | 1.012 (0.988 – 1.038) | 0.33 |
| **PM_10_ Threshold-modelled, >50 µg/m³** | | | | | | | | |
| **All** | 1.006 (0.999 – 1.014) | 0.07 | 1.004 (0.997 – 1.012) | 0.24 | 1.006 (0.999 – 1.014) | 0.11 | 1.011 (1.002 – 1.021) | 0.020 |
| **A/B/AB** | 1.005 (0.997 – 1.014) | 0.22 | 1.004 (0.995 – 1.013) | 0.40 | 1.006 (0.997 – 1.015) | 0.22 | 1.006 (0.995 – 1.018) | 0.26 |
| **O** | 1.012 (0.999 – 1.025) | 0.06 | 1.009 (0.995 – 1.022) | 0.20 | 1.010 (0.996 – 1.024) | 0.16 | 1.026 (1.009 – 1.044) | 0.003 |
| • Data presented in parentheses are 95% CI.  • Abbreviations: MA – moving average; OR – odds ratio; , CI – confidence interval  • Values have been scaled so that each term's OR and 95% CI are representative of an increase of +10 µg/m³. | | | | | | | | |
