# Supplementary material for: Associations of air pollution with acute coronary syndromes based on A/B/AB versus O blood types: case-crossover study
Source: Sci Rep. 2024 Jun 25;14:14580. doi: 10.1038/s41598-024-65506-2 (PMC11199661; doi:10.1038/s41598-024-65506-2)
Supplement: Supplementary file 7 — Supplementary Table S3. [file 41598_2024_65506_MOESM7_ESM.docx]

| **Table S3. Results of conditional logistic regression for PM2.5 and PM10 without (base model) and with adjustment (TH and THWN models) for weather variables in a subset of individuals with weather data available (2012-2015 only).** | | | | | | | | |
| --- | --- | --- | --- | --- | --- | --- | --- | --- |
|  |  | | | | | | | |
|  | **1-day lag** | | **2-day MA** | | **3-day MA** | | **7-day MA** | |
| **Blood type** | **OR (95% CI)** | **p value** | **OR (95% CI)** | **p value** | **OR (95% CI)** | **p value** | **OR (95% CI)** | **p value** |
| **Analyses of PM_2.5_**  **Threshold-modelled (PM_2.5_ >35 µg/m³)** | | | | | | | | |
| **All (base)** | 1.011 (0.997 – 1.026) | 0.12 | 1.016 (1.012 – 1.032) | 0.030 | 1.010 (0.995 – 1.026) | 0.20 | 1.008 (0.990 – 1.027) | 0.39 |
| **All (TH)** | 1.008 (0.993 – 1.022) | 0.29 | 1.011 (0.995 – 1.026) | 0.17 | 1.004 (0.987 – 1.020) | 0.67 | 1.001 (0.982 – 1.021) | 0.89 |
| **All (THWN)** | 1.010 (0.995 – 1.025) | 0.20 | 1.015 (0.998 – 1.032) | 0.09 | 1.007 (0.989 – 1.024) | 0.46 | 1.001 (0.981 – 1.021) | 0.93 |
| **Non-threshold PM_2.5_** | | | | | | | | |
| **All (base)** | 1.014 (0.995 – 1.032) | 0.15 | 1.016 (0.996 – 1.037) | 0.11 | 1.013 (0.991 – 1.036) | 0.24 | 1.019 (0.990 – 1.048) | 0.21 |
| **All (TH)** | 1.010 (0.991 – 1.029) | 0.31 | 1.010 (0.990 – 1.031) | 0.34 | 1.006 (0.984 – 1.030) | 0.58 | 1.013 (0.984 – 1.042) | 0.39 |
| **All (THWN)** | 1.013 (0.993 – 1.033) | 0.20 | 1.016 (0.993 – 1.040) | 0.18 | 1.011 (0.986 – 1.036) | 0.40 | 1.014 (0.984 – 1.045) | 0.36 |
| **Threshold-modelled (PM_2.5_ >25 µg/m³)** | | | | | | | | |
| **All (base)** | 1.012 (0.997 – 1.026) | 0.11 | 1.010 (0.995 – 1.026) | 0.20 | 1.007 (0.991 – 1.024) | 0.38 | 1.011 (0.992 – 1.031) | 0.27 |
| **All (TH)** | 1.008 (0.993 – 1.023) | 0.28 | 1.006 (0.990 – 1.022) | 0.49 | 1.003 (0.986 – 1.020) | 0.75 | 1.008 (0.989 – 1.028) | 0.42 |
| **All (THWN)** | 1.011 (0.995 – 1.026) | 0.18 | 1.010 (0.992 – 1.028) | 0.26 | 1.007 (0.988 – 1.026) | 0.47 | 1.009 (0.989 – 1.030) | 0.36 |
| **Analyses of PM_10_**  **Threshold-modelled (PM_10_ >150 µg/m³)** | | | | | | | | |
| **All (base)** | 1.022 (1.002 – 1.042) | 0.030 | 1.006 (0.987 – 1.025) | 0.55 | 1.022 (0.989 – 1.057) | 0.19 | Not available | ----- |
| **All (TH)** | 1.022 (1.002 – 1.042) | 0.033 | 1.004 (0.985 – 1.024) | 0.67 | 1.022 (0.988 – 1.057) | 0.21 | Not available | ----- |
| **All (THWN)** | 1.022 (1.002 – 1.043) | 0.028 | 1.007 (0.987 – 1.027) | 0.51 | 1.022 (0.988 – 1.058) | 0.20 | Not available | ----- |
| **Non-threshold PM_10_** | | | | | | | | |
| **All (base)** | 1.009 (0.996 – 1.022) | 0.18 | 1.011 (0.996 – 1.025) | 0.15 | 1.013 (0.997 – 1.029) | 0.12 | 1.021 (1.0001 – 1.042) | 0.048 |
| **All (TH)** | 1.009 (0.996 – 1.022) | 0.18 | 1.010 (0.995 – 1.024) | 0.19 | 1.012 (0.996 – 1.028) | 0.14 | 1.023 (1.002 – 1.044) | 0.032 |
| **All (THWN)** | 1.010 (0.997 – 1.024) | 0.14 | 1.013 (0.997 – 1.030) | 0.12 | 1.016 (0.998 – 1.033) | 0.08 | 1.025 (1.003 – 1.046) | 0.024 |
| **Threshold-modelled (PM_10_ >50 µg/m³)** | | | | | | | | |
| **All (base)** | 1.007 (0.997 – 1.016) | 0.15 | 1.007 (0.997 – 1.017) | 0.15 | 1.010 (0.9998 – 1.021) | 0.055 | 1.012 (1.0001 – 1.024) | 0.048 |
| **All (TH)** | 1.006 (0.997 – 1.016) | 0.19 | 1.006 (0.996 – 1.017) | 0.22 | 1.009 (0.999 – 1.020) | 0.08 | 1.013 (1.001 – 1.025) | 0.039 |
| **All (THWN)** | 1.007 (0.997 – 1.017) | 0.17 | 1.008 (0.997 – 1.019) | 0.16 | 1.011 (0.9999 – 1.023) | 0.052 | 1.013 (1.001 – 1.026) | 0.038 |
| • Data presented in brackets are 95% CI. • Abbreviations: base – models entered PM_2.5_ or PM_10_ measurements only; MA – moving average; OR – odds ratio; TH – models adjusted for temperature and humidity; THWN – models adjusted for temperature, humidity, wind speed, and nebulosity • Values have been scaled so that each term's OR and 95% CI are representative of an increase of +10 µg/m³. • For threshold-modelled predictors all PM_2.5_ or PM_10_ values less than the threshold were set to 0. | | | | | | | | |
